# Current and emerging drug targets in heart failure treatment

**DOI:** 10.1007/s10741-021-10137-2

**Published:** 2021-07-17

**Authors:** Nicolò Ghionzoli, Francesco Gentile, Anna Maria Del Franco, Vincenzo Castiglione, Alberto Aimo, Alberto Giannoni, Silvia Burchielli, Matteo Cameli, Michele Emdin, Giuseppe Vergaro

**Affiliations:** 1 Department of Medical Biotechnologies, Division of Cardiology, University of Siena, Siena, Italy; 2 Cardiology Division, University Hospital of Pisa, Pisa, Italy; 3 Division of Cardiology and Cardiovascular Medicine, Fondazione Toscana Gabriele Monasterio, Via Moruzzi, 1 - 56124 Pisa, Italy; 4 Institute of Life Sciences, Scuola Superiore Sant’Anna, Pisa, Italy; 5 Fondazione Toscana Gabriele Monasterio, Pisa, Italy

**Keywords:** Heart failure, Pharmacotherapy, Pharmacodynamics, Neurohormonal antagonism, Emerging targets, SGLT2 inhibitors

## Abstract

After initial strategies targeting inotropism and congestion, the neurohormonal interpretative model of heart failure (HF) pathophysiology has set the basis for current pharmacological management of HF, as most of guideline recommended drug classes, including beta-blockers, angiotensin-converting enzyme inhibitors, angiotensin receptor blockers, and mineralocorticoid receptor antagonists, blunt the activation of detrimental neurohormonal axes, namely sympathetic and renin–angiotensin–aldosterone (RAAS) systems. More recently, sacubitril/valsartan, a first-in-class angiotensin receptor neprilysin inhibitor, combining inhibition of RAAS and potentiation of the counter-regulatory natriuretic peptide system, has been consistently demonstrated to reduce mortality and HF-related hospitalization. A number of novel pharmacological approaches have been tested during the latest years, leading to mixed results. Among them, drugs acting directly at a second messenger level, such as the soluble guanylate cyclase stimulator vericiguat, or other addressing myocardial energetics and mitochondrial function, such as elamipretide or omecamtiv-mecarbil, will likely change the therapeutic management of patients with HF. Sodium glucose cotransporter 2 inhibitors, initially designed for the management of type 2 diabetes mellitus, have been recently demonstrated to improve outcome in HF, although mechanisms of their action on cardiovascular system are yet to be elucidated. Most of these emerging approaches have shifted the therapeutic target from neurohormonal systems to the heart, by improving cardiac contractility, metabolism, fibrosis, inflammation, and remodeling. In the present paper, we review from a pathophysiological perspective current and novel therapeutic strategies in chronic HF.

## Introduction

Heart failure (HF) represents a global health issue, as it is estimated to affect 1–2% of the general population, with an even higher prevalence in cohorts of elderly subjects [1]. Several interpretative models have been proposed over the last decades to explain HF pathophysiology. They initially focused on the concomitant impairment of both heart and kidney (cardio-renal model), and later on hemodynamic alterations secondary to pump failure (hemodynamic model) [2]. In the last 30 years, the neurohormonal model has taken a central role, following the demonstration that derangement of neurohormonal activation and of peripheral feedbacks acts as promoter in HF syndrome and represents the pathophysiological basis for the use of most of pharmacological classes with a prognostic benefit [3].

**Fig. 1:**
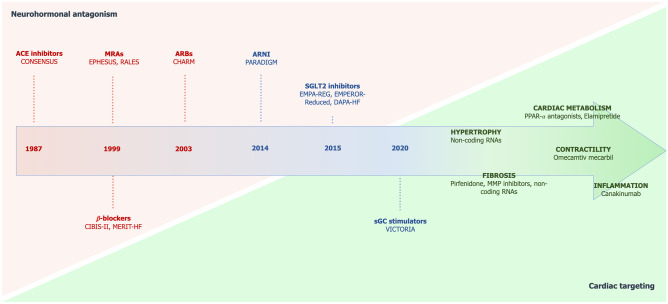
Chronologic development of drugs in heart failure, highlighting the shift from neurohormonal antagonism to specific cardiac targeting. Current guideline recommended drugs classes are represented in bold red; drug classes with existing evidence on an outcome benefit in heart failure are represented in bold blue; novel possible targets are represented in green. *ACE* angiotensin-converting enzyme; *ARBs* angiotensin receptor blockers; *ARNI* angiotensin receptor neprilysin inhibitor; *MRAs* mineralocorticoid receptor antagonists; *sGC* soluble guanylate cyclase; *SGLT2* sodium-glucose cotransporter 2

In clinical practice, HF is usually classified based on left ventricular ejection fraction (LVEF) into HF with reduced ejection fraction (HFrEF, LVEF < 40%), HF with preserved ejection fraction (HFpEF, LVEF ≥ 50%), and in the recently proposed category of HF with mid-range ejection fraction (HFmrEF, LVEF 40–49%) [4]. While many pharmacological and non-pharmacological therapies of HFrEF have shown a prognostic benefit, the outcome of patients with HFrEF still remains poor [5]. To date, few evidence-based data are available as concerns treatment of patients with milder degree of LV systolic dysfunction, as the majority of the drugs investigated have failed to demonstrate prognostic benefit in HFpEF [6]. Nevertheless, novel therapies are emerging in latest years, most of them targeting myocardial energetics, shifting the focus back from the periphery to the heart muscle (Fig. 1). Herein, we review the current and novel potential therapeutic strategies in chronic HF from a pathophysiological perspective.

## Current therapeutic targets

### Sympathetic nervous system

**Fig. 2:**
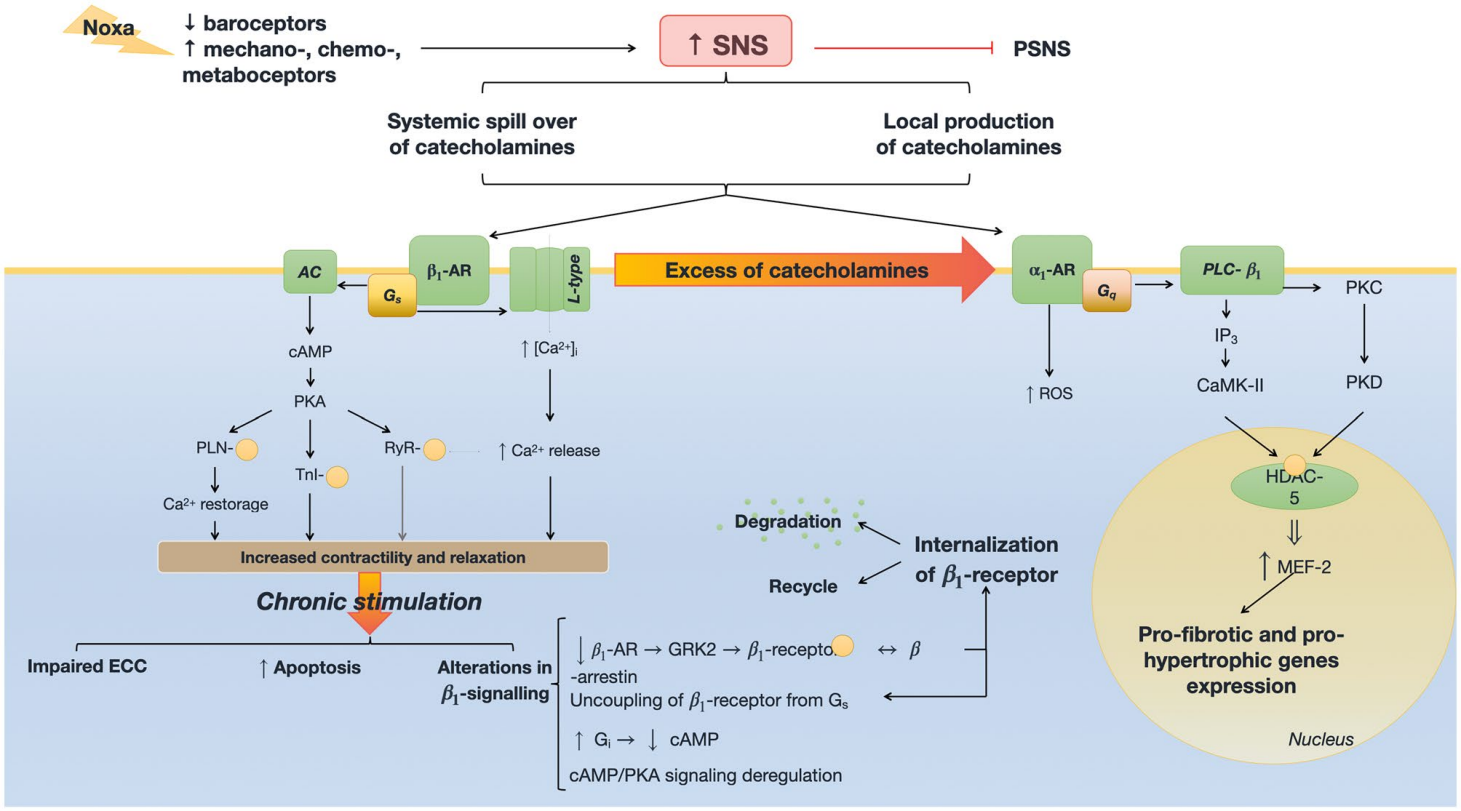
Molecular signaling of sympathetic nervous system (SNS) activation in the cardiomyocyte in heart failure. ${\alpha }_{1}$*-AR* ${\alpha }_{1}$ adrenergic receptor; *AC* adenylate cyclase; *AR* adrenergic receptor; *CaMK-II* calcium-calmodulin kinase type 2; *cAMP* cyclic adenosine monophosphate; *Gi* G-protein “i” associated with trans-membrane receptor; *Gq* G-protein “q” associated with trans-membrane receptor; *GRK2* complex G protein-coupled receptor kinase type 2; *Gs* G-protein “s” associated with trans-membrane receptor; *HDAC-5* histone deacetylase type 5; *IP3* inositol trisphosphate; *L-type* L-type calcium channel; *MEF-2* myocyte enhancer factor type 2; *PKA* protein-kinase A; *PKC* protein-kinase C; *PKD* protein-kinase D; *PLC-*${\beta }_{1}$ phospholipase C-${\beta }_{1}$; *PLN* phospholamban; *PSNS* parasympathetic nervous system; *ROS* reactive oxygen species; *RyR* ryanodine receptor; *SR* sarcoplasmic reticulum; *TnI* troponin-I

HF is characterized by an imbalance between sympathetic and parasympathetic afferent systems. A blunted baroreflex is usually combined with hyperactive chemo- and ergoreflexes [7, 8]. The final result of this imbalance is an overactivation of the sympathetic nervous system (SNS), initially aimed at restoring the circulatory homeostasis. Chronic stimulation of SNS causes a systemic spill-over of catecholamines, attributed to their increased release and reduced reuptake, and to an excessive intra-myocardial production [9] (Fig. 2).

Stimulation of $\beta$ 1 adrenergic receptor by catecholamines, mainly by norepinephrine, activates G_S_ protein [10]. G_S_ protein in turn stimulates L-type calcium channels with increased calcium conductance, and activates adenylate-cyclase, thus resulting in an increased cyclic adenosine monophosphate (cAMP) production and in protein-kinase A activation. Protein-kinase A regulates phosphorylation of phospholamban, troponin I, and ryanodine receptor, thus improving myocardial contraction and relaxation [11]. Chronic stimulation of adrenergic receptors holds detrimental effects. The role of α1 receptors is less defined, and there is evidence that they may hold important adaptive functions, promoting cardiomyocyte survival and protecting against adverse remodeling [12]. Conversely, persistent activation of $\beta$ 1-adrenergic receptor negatively affects excitation–contraction coupling (ECC) and enhances pro-apoptotic pathways [13] (see also Table 1).

**Table 1:** Main molecular pathways involved in heart failure (HF) pathophysiology, therapeutic targets, second messengers, adaptive, and maladaptive effects on the cardiovascular system and related drugs. Entries in italics represent the current guidelines-based drugs for heart failure. Entries in bold represent drugs with demonstrated benefits in the outcome of heart failure, yet not included in guidelines. *AC* adenylate cyclase; *cAMP* cyclic adenosine monophosphate; *cGMP* cyclic guanosine monophosphate; *CPT* Acetyl-CoA C-acetyl transferase; *DAG* diacyl-glycerol; *ET* endothelin receptor; *HIF* hypoxia inducible factor; *HSD* hydroxysteroid dehydrogenase; *ICAM* intercellular adhesion molecule 1; *IL* interleukin; *IP3* inositol triphosphate; *JAK/STAT* Janus kinase/signal transducer and activator transcription factor; *LDLox* oxidized LDL; *MEF* myocyte enhancer factor; *MMP* metalloproteinases; *NADPH* nicotinamide-adenine dinucleotide; *NO* nitric oxide; *NP* natriuretic peptide; *NPR* natriuretic peptide-binding receptor; *PKA* protein kinase A; *PKB* protein kinase B; *PKC* protein kinase C; *PKG* protein kinase G; *PLC* phospholipase; *PlGF* placental-derived growth factor; *PP-* protein phosphatase-; *PPAR* peroxisome proliferator-activated receptor; *PTP* phosphotyrosine phosphatase; *ROS* reactive oxygen species; *RyR* ryanodine receptor; *SERCA* sarco/endoplasmic reticulum calcium ATPase; *TGF* transforming growth factor; *TRPV* transient receptor potential cation channel subfamily V; *vSMCs* vascular smooth muscle cells

Pathway	Receptor/target	Second messenger	Adaptive effects	Maladaptive effects	Drugs
Sympathetic nervous system	${\beta }_{1}$	G_s_ > AC > cAMP > PKA	Increased contractility and relaxation	Excitation contraction uncoupling Apoptotic pathways Alterations in ${\beta }_{1}$ signals	*Selective *$\beta$* blockers (e.g., bisoprolol)*
	${\alpha }_{1}$	G_q_ > PLC-${\beta }_{1}$ > DAG, IP3, PKC > MEF-2 ROS		Pro-fibrotic and pro-hypertrophic genes expression	*Non-selective *$\beta$* blockers (e.g., carvedilol)*
Renin–angiotensin–aldosterone system	AT-1	G_q_ > NADPH oxidase – ROS JAK/STAT > PTK PLC > DAG, IP3, PKC Tyrosine kinase—MAPK		Vasoconstriction, inflammation, proliferation, atherosclerosis Inflammation, growth, proliferation DAG, IP3 > vasoconstriction Inflammation, growth, proliferation	*ACE inhibitors, AT-1 antagonists (ARBs)*
	AT-2	Bradykinins > NO > cGMP G_q_ > PP2A, PTP > ↓ MAPK	Vasodilation, blunt in inflammation, growth and proliferation		*ACE inhibitors*
	MR	Tissues with 11-$\beta$-HSD2: Kidney: sodium-water retention vSMCs: galectin-3; PKB; PlGF Endothelium: ICAM. Tissues without 11-$\beta$-HSD2: Cardiomyocytes Macrophages: M1 phenotype	Increased contractility	Hypertension Fibrosis; apoptosis; atherosclerosis Leukocytes adhesion Hypertrophy, electric instability, oxidative stress Fibrosis and damage	*MR antagonists (MRAs)*
Natriuretic peptides	NPR-A	GC > cGMP > PKG	Vasodilation, diuresis, natriuresis, inhibition of cardiac hypertrophy and remodeling, suppression of ADH, blunt in SNS discharge		*Angiotensin receptor/Neprilysin inhibitor*
	NPR-B		Inhibition of vSMC proliferation, LDLox migration, ET-1 release		
	NPR-C	Internalization of NPs for degradation		Blunt in NPs effects	
Nitric oxide	Guanylate cyclase	cGMP	Vasodilation and muscular relaxation		**Soluble guanylate cyclase stimulators (vericiguat) and activators**
SGLT-2	SGLT-2	Glucose and sodium reabsorption		Glucose, sodium and water retention Activation of SNS? Oxidative stress? Cardiomyocyte metabolic impairment with usage of unfavorable substrates?	**Empagliflozin, Dapagliflozin**
Endothelin	ET-A	G_q_ > PLC > DAG, IP3 G_s_	Increased contractility	Fibrosis, arrhythmias, hypertrophy, vasoconstriction	Endothelin receptor antagonists, both selective (-A or -B) or non-selective
	ET-B	G_q_ > PLC > DAG, IP3 G_s_	cGMP-dependent endothelium-mediated vasodilation	Fibrosis, apoptosis, hypertrophy, PKC-dependent vasoconstriction	
Anti-diuretic hormone	V1a	G_q_ > PLC > DAG, IP3	Increased contractility	Vasoconstriction, platelets aggregation, hypertrophy	Vaptans
	V1b			Increased ACTH production	
	V2	G_s_ > AC > cAMP > aquaporin 2		Water reabsorption, hyponatremia	
Mitochondria	PPAR-$\alpha$	Transactivation of genes for $\beta$-oxidation: CPT-1, MCAD		Induces shift to lipidic metabolism	PPAR-$\alpha$ antagonists?
	CPT			Stimulation of $\beta$-oxidation	Trimetazidine
	Cardiolipin	Mitochondrial membrane stabilizer	Efficient mitochondrial respiration		Elamipretide?
	Q10 coenzyme		Efficient mitochondrial respiration		Q10 supplements
Nox2 ROS	↓ PP-1 activity		Increased activity of SERCA and RyR	Impairment of the activity of both proteins, due to chronic oxidation	Antioxidants? Elamipretide?
Nox4 ROS	↓ PP-1 activity HIF stabilized		Angiogenesis	Hypertrophy	
Inflammation	e.g. IL-1				Canakinumab?
Fibrosis	TGF-$\beta$			Cardiac fibrosis	Pirfenidone?
	MMP				MMP inhibitors?
	Galectin-3				Antisense RNA?
TRPV-4	TGF-${\beta }_{1}$	Calcium	Vasodilation, arteriogenesis	Cardiac fibrosis, pulmonary hypertension, HF-related pulmonary edema	TRPV-4 inhibitors?

Inhibition of adrenergic receptors is effectively achieved by $\beta$-blockers, a wide and heterogeneous class of drugs. Their use in HF has been firstly debated because of their negative chronotropic and inotropic effects. Actually, their use has been proved effective, and represents a cornerstone of the pharmacological treatment for HF [14]. Specifically, $\beta$-blockers cause a reduction in myocardial oxygen consumption and prevent the detrimental consequences of a longstanding adrenergic stimulation [15]. Some molecules can also inhibit $\alpha$ adrenergic receptor (non-selective $\beta$-blockers, such as carvedilol); other can exert a weak agonism to the $\beta$-receptor (i.e., pindolol), a property known as intrinsic sympathomimetic activity, but none of them has been proven effective in HF. $\beta$-blockers, particularly non-selective ones, might cause subtype-specific upregulation of adrenergic receptors and therefore should not be withdrawn abruptly [16].

### Renin–angiotensin–aldosterone system

#### Renin and angiotensins

Renin–angiotensin–aldosterone system (RAAS) is one of the main drivers in HF pathophysiology. Renin release from juxtaglomerular apparatus is the first step in the RAAS cascade. SNS activation, together with renal hypoperfusion and reduced filtered sodium levels reaching the *macula densa* are the main stimuli for renin secretion [13, 17]. Renin converts angiotensinogen—synthetized by the liver—into Ang-I, which is further cleaved into Ang-II by the angiotensin-converting enzyme (ACE), and, to a lesser extent, by other enzymes, such as chymases [18]. Renin, as well as its precursor prorenin, also exists as a cytosolic protein with other non-enzymatic functions [19]. The cytosolic renin exerts different and even opposite functions to those of secretory renin; specifically, whereas secretory renin promotes necrosis and fibrosis, the cytosolic renin variant protects cells from necrotic death. Binding of either secretory renin or prorenin to its (pro)renin receptor triggers downstream intracellular signals leading to the overexpression of pro-fibrotic genes. The interaction between renin and (pro)renin receptor also increases renin cleavage activity at cell surface [20].

**Fig. 3:**
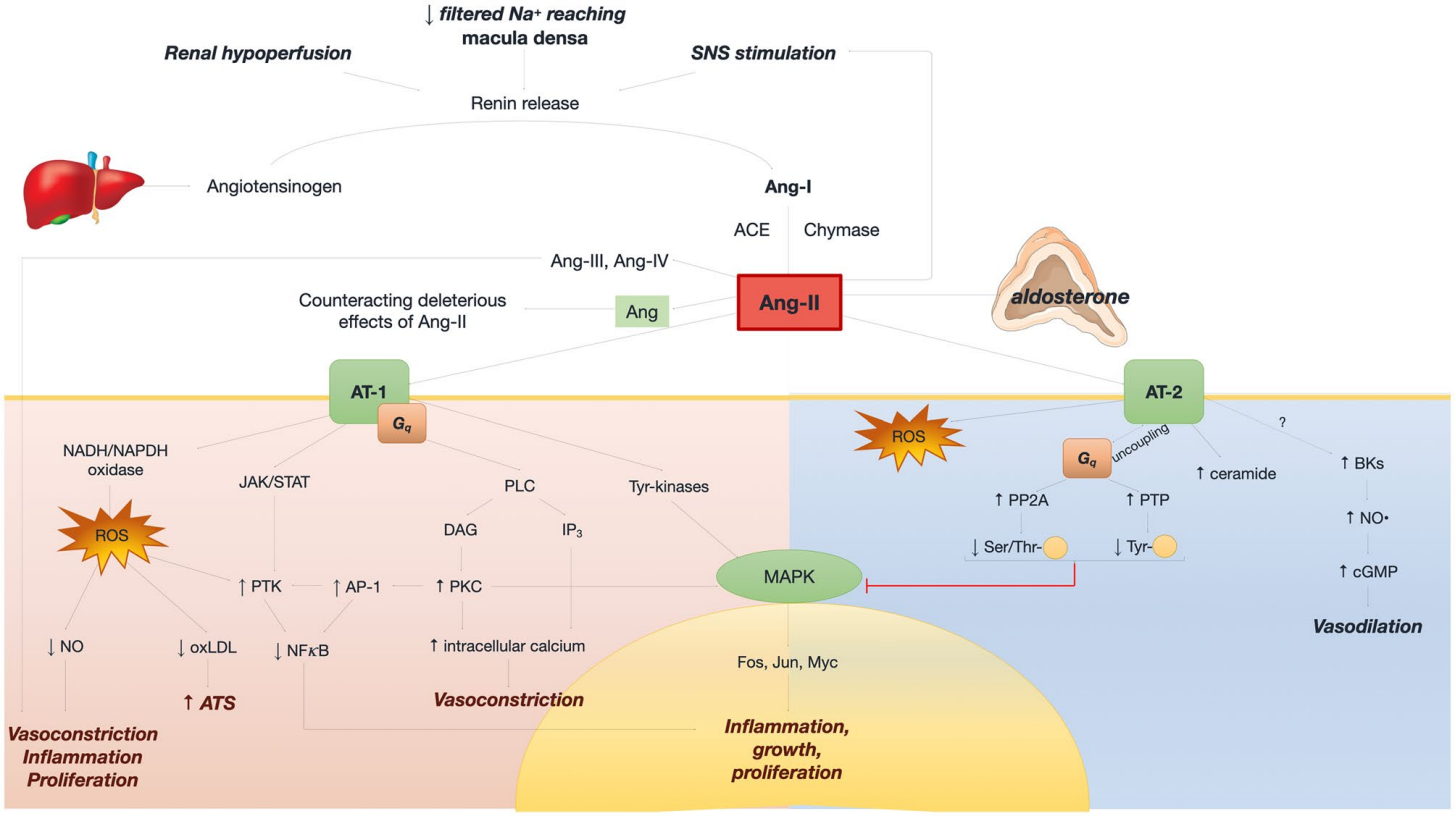
Molecular signaling of the renin–angiotensin–aldosterone system in heart failure. Angiotensin receptor 1 (AT-1) as well as many angiotensins (II, III, IV) are responsible for vasoconstriction, inflammation, proliferation and atherosclerosis. AT-2 counteracts these detrimental responses mainly via vasodilation. *Ang* angiotensin; *AP-1* activator protein 1; *ATS* atherosclerosis, *BK* bradykinin; *cGMP* cyclic guanosine monophosphate; *DAG* diacyl-glycerol; *IP3* inositol-triphosphate; *JAK/STAT* Janus kinase/signal transducer and activator transcription factor; *NAD(P)H* nicotinamide-adenine dinucleotide (phosphate); *NFkB* nuclear factor kappa-*B*; *NO* nitric oxide; *oxLDL* oxidized low-density lipoprotein; *PKC* protein kinase C; *PLC* phospholipase; *PP2A* protein phosphatase 2A; *PTK* phosphotyrosine kinase; *PTP* phosphotyrosine phosphatase; *Ser/Thr* serine/threonine; *Tyr* tyrosine

Ang-II interacts with angiotensin receptor (AT) 1 and AT-2. Ang-II directly regulates glomerular homeostasis and stimulates aldosterone production in the adrenal gland. Downstream actions following the interaction of Ang-II with AT-1 include nicotinamide-adenine dinucleotide phosphate (NADPH) oxidase activation, JAK-STAT signaling, phospholipase C pathway, and tyrosine kinases activation [21, 22]. NADPH oxidase activation causes ROS production, reduced nitric oxide (NO) concentration, inflammation, and proliferation [23–25]. Ang-II/AT-2 interaction promotes ROS production, increases intracellular ceramide levels, and causes the uncoupling of AT-2 from G_q_, leading to a reduction of mitogen-activated protein kinase (MAPK) activity and of inflammatory, proliferative, and growth-related effects [26]. Ang-II is also cleaved into other peptides (Ang-III and Ang-IV), causing vasoconstrictive effects, and Ang peptide 1–7 via ACE isoform 2, counteracting the deleterious effects of Ang-II [27] (Fig. 3). Ang 1–7 also have direct cardioprotective and vasodilatory actions, by inducing the release of NO and prostaglandins and by antagonizing AT-1 [28, 29]. Indeed, preclinical studies have shown that Ang 1–7 blunts ischemia–reperfusion injury and inhibits Ang-II-induced cardiac hypertrophy and remodeling [30, 31]. The latter effect is mostly mediated by the binding to its high selective G-protein coupled Mas receptor [32].

RAAS blockade plays a key role in the neurohormonal antagonism in HF. Many molecules targeting different steps in the RAAS cascade have been evaluated, with ACE inhibitors, AT-1 receptor blockers (ARBs), and aldosterone antagonists being the most widely tested. ACE inhibitors and ARBs have been introduced in clinical practice as potent vasodilators, but also showed antiremodeling properties in the setting of either ischemic and non-ischemic LV dysfunction [33–35].

Compared to ACE inhibitors, ARBs have a downstream activity as they prevent Ang-II from binding to AT-1. This was hypothesized to contribute to a binding shift of Ang-II to AT-2, thus resulting in additional antifibrotic effects vs. ACE inhibitors. Nonetheless, there is weaker evidence on the clinical efficacy of ARBs compared to ACE inhibitors in HF patients [4]. Although a potential advantage from a double RAAS blockade with a combination of ACE inhibitors and ARBs may be envisaged, such strategy did not prove effective in clinical practice. Further, the direct renin inhibitor aliskiren did not reduce the rates of all-cause and cardiovascular mortality in HF patients [36]. Novel renin inhibitors are currently under investigation and are showing promising results in murine models [37]. Finally, as discussed in a following paragraph, combined angiotensin receptor and neprilysin inhibition has become the first option among drugs acting on RAAS.

Vasodilation as a potential therapeutic target has been addressed also before RAAS inhibition, as hydralazine—an inositol triphosphate inhibitor—reduced mortality by 34% in the V-HeFT trial [38]. Today, it is used especially in the Afro-Americans, as it showed a 47% reduction in mortality in the A-HeFT trial [39].

#### Aldosterone

Aldosterone is a steroid hormone produced by the adrenal cortex. Aldosterone signaling includes genomic and non-genomic actions, which are mediated by intracellular and membrane mineralocorticoid receptors (MRs), respectively [40]. Although the intracellular receptor is responsible for the potential binding of several molecules, including aldosterone and glucocorticoids, the receptor specificity for aldosterone depends on the presence of 11-$\beta$-hydroxysteroid dehydrogenase 2 [41]. The intracellular MR mediates sodium-water retention in renal epithelial cells of the distal nephron, stimulates fibrosis, apoptosis, and atherosclerosis in vascular smooth muscle cells (vSMCs) and promotes leukocyte adhesion, pro-thrombotic phenotype and epithelial–mesenchymal transition in endothelial cells [42].

The intracellular MR is also present in other non-epithelial tissues, acting in a 11-$\beta$-hydroxysteroid dehydrogenase 2-independent fashion [43]. In macrophages, MR activation promotes M_1_-phenotype differentiation, thus leading to galectin-3 secretion, fibroblast activation, and deposition of fibrous tissue [44]. In cardiomyocytes, MR usually acts as glucocorticoid receptor, and increases contractility, stimulates hypertrophy, and contributes to electrical remodeling [45].

While MR antagonists (MRAs) were first employed in clinical practice as potassium-sparing diuretics, they have been proven useful in cardiovascular diseases and in HF, mostly due to their antifibrotic and antihypertrophic actions [46]. Currently available molecules have either a steroidal (i.e., spironolactone and eplerenone) or non-steroidal structure (i.e., finerenone). The in-class and between-class differences mainly stand in the selectivity (rather than in the affinity) for MR, as only steroidal MRA can also bind to other steroids receptors. As the founder of this class, spironolactone has a similar structure to both aldosterone and progesterone, whereas eplerenone more specifically binds MR. This results in a reduced incidence of adverse drug reactions related to the binding to sexual hormones receptors [47]. Finerenone shows the highest specificity within this class, and is currently under a phase-III clinical trial evaluation for the treatment of HF [48].

MR blockade, as well as the use of ACE inhibitors and ARBs, has been associated with a rebound increase in circulating aldosterone, named the aldosterone breakthrough [49]. Although the mechanisms underlying this phenomenon are still to be clarified, the ACE-independent synthesis of RAAS effectors has been advocated.

### Endothelin

Endothelin plays pleiotropic roles in the cardiovascular system. Synthesis of pre-pro-endothelin is stimulated by several neuro-hormones (i.e., Ang-II, norepinephrine), cytokines (i.e. interleukin-1), hypoxia, acidosis and shear stress [50]. The precursor is then cleaved by proteases into pro-endothelin, which is further processed into endothelin, the biologically active form, by the endothelin-converting enzyme [51].

Endothelin binds to receptors ET-A and ET-B, both expressed in heart and vessels. ET-A plays a major role on cardiomyocytes, mediating hypertrophy, fibrosis, and favoring the onset of arrhythmias; it also exerts a positive inotropic effect by increasing intracellular calcium concentration [52, 53]. In blood vessels, ET-A promotes fibrosis through fibroblast activation and mediates vasoconstriction in vSMCs via G_q_-phospholipase C signaling [54]. Furthermore, it has been demonstrated that ET-A is regulated by complex G protein-coupled receptor kinase type 2-mediated phosphorylation that may increase its affinity for arrestins [55]. ET-B expression is higher in fibroblast than in cardiomyocytes, promoting fibrosis, apoptosis, and hypertrophy [56]. In the vasculature, ET-B is responsible for fibrosis, acting synergistically with ET-A, as well as for vasoconstrictive signals in vSMCs via protein kinase C [57].

Despite the role of endothelin in the progression of end-organ damage, many drugs antagonizing ET-A and/or ET-B (i.e., bosentan, sitaxentan) displayed no beneficial effects compared to placebo, while some others were shown to be harmful, both in the settings of acute and chronic HF [58–60].

### Antidiuretic hormone

The antidiuretic hormone (ADH) pathway has been considered one of the leading pathophysiological drivers of HF, especially in advanced stages, when hyponatremia is more often observed [61]. Despite this assumption, the main clinical trials targeting ADH system have yielded controversial results, mainly with non-significant improvements in hard endpoints [62].

In physiological conditions, ADH is produced by the supraoptic and paraventricular nuclei in the hypothalamus and then stored in the neurohypophysis. Its release mainly depends on osmotic imbalance, sensed by specific receptors. In patients with HF, ADH secretion is also dependent on non-osmotic mechanisms, especially SNS and RAAS overactivation.

ADH acts on three types of receptors: V1a, V1b, V2 [63]. V1a signaling is mediated by G_q_-PLC pathway, thus inducing vSMCs vasoconstriction, platelet aggregation, and myocyte modifications, such as activation of growth factors, synthesis of contractile proteins and positive inotropic effect. The binding of V1b causes an increased production of adrenocorticotropic hormone by the adenohypophysis [64]. Finally, V2 stimulation in the kidney increases the exposure of aquaporin 2 at the plasma membrane, which is in turn is responsible for a higher rate of free water reabsorption, plasma dilution, and hyponatremia [65].

Conivaptan is a non-selective, intravenous V1a and V2 antagonist, whereas tolvaptan is a selective V2 antagonist which has been shown to ameliorate serum sodium levels in acute and chronic HF [66, 67]. Both drugs target free water retention and have been proposed as second-line diuretics [68], whilst conivaptan has further antiremodeling properties. Nevertheless, both these molecules failed to significantly reduce mortality in clinical studies [69].

### Natriuretic peptides

The natriuretic peptide system represents the main counterregulatory axis, with diuretic, natriuretic, vasodilative, and antifibrotic actions [70–72]. The main stimulus to the release of natriuretic peptides is the increase in cardiac wall tension. Atrial natriuretic peptide (ANP), produced mainly by atrial cardiomyocytes, is released following acute changes in atrial wall stress, whereas B-type natriuretic peptide (BNP) secretion is finely, transcriptionally regulated by a chronic overload of the left ventricle [73, 74]. Moreover, several cardiac (e.g. atrial fibrillation) or extracardiac co-factors (such as pulmonary comorbidities, renal function, age and body mass index) might affect circulating levels of natriuretic peptide [75].

Pro-ANP, cleaved by pre-pro-ANP, is a peptide precursor enzymatically processed by corin into the inactive fragment N-terminal (NT)-pro-ANP and into the active peptide ANP [76]. ANP release is promoted by a large number of damage systems, including endothelin, Ang-II, and ADH [77]. BNP is produced by the enzymatic cleavage of proBNP (derived from pre-proBNP) by corin and furin. BNP is released with the biologically inactive N-terminal fragment (NT-proBNP) in an equimolar fashion [78]. Both ANP and BNP can bind to two types of membrane receptors: natriuretic peptide receptor (NPR)-A, which is responsible for their biological actions, and NPR-C, which is involved in the internalization and the degradation of natriuretic peptides. BNP can further bind to NPR-B [79]. NPR-A and NPR-B stimulation increases the activity of guanylate cyclase (GC), which turns into an increase in cyclic guanosine monophosphate (cGMP) production and protein-kinase G activation [80]. Following protein kinase G activation, natriuretic peptides induce renal vasodilation, natriuresis, and diuresis via dilation of the afferent and constriction of the efferent arteriole, reduce sodium-water reabsorption and inhibit RAAS and ADH signaling pathways. Other targets include the increase in parasympathetic stimulation and the blunting of sympathetic activity, as well as the inhibition of fibroblasts, macrophages, pro-inflammatory cytokines, and pro-hypertrophic stimuli [71, 72, 77].

C-type natriuretic peptide (CNP) is produced by pre-proCNP following a double enzymatic cleavage in brain, endothelial cells, heart, fibroblasts, and macrophages [75, 81]. CNP binds to NPR-B and, by increasing cGMP levels, inhibits vSMCs proliferation, oxidized low-density lipoprotein accumulation in the arterial walls, endothelin release, phosphorylation of calmodulin kinase, and extracellular signal-regulated kinase [82].

Plasma levels of natriuretic peptides are dependent on both synthesis and clearance processes. NPR-C is the third isoform of natriuretic peptides receptors, whose binding determines the internalization of the ligand-receptor complex and the ligand degradation [83]. Neprilysin is a zinc-dependent endopeptidase, which is able to degrade all natriuretic peptides, although with a higher affinity for ANP than for BNP [84]. Neprilysin has also other substrates, including Ang-II, glucagon, and bradykinin [85].

Despite their important counterbalancing role, natriuretic peptides can fully oppose detrimental drivers only at earlier stages of disease. Along with HF progression, multiple derangements in natriuretic peptide system occur, including a reduced expression of corin and furin leading to low biologically active peptides, an increased dipeptidyl-peptidase IV activity causing the rise in circulating levels of truncated pro-BNP and BNP, a downregulation of NPR-A and NPR-B, a desensitization of NPR-A/GC system and an increase NPR-C-mediated clearance [86].

The first attempt in targeting this system was to administer exogenous natriuretic peptides. Despite initial optimism, the largest trial on nesiritide (a recombinant form of BNP) failed to show a difference in mortality or re-hospitalization rates versus placebo, and disclosed poor tolerability (e.g., hypotension and worsening renal function), leading to approval withdrawal [87, 88]. More stable molecules have been developed and are currently undergoing clinical tests. M-ANP, a natriuretic peptide analogue, was shown to improve natriuresis and glomerular filtration rate as compared with endogenous ANP [89].

Many attempts have been made to test neprilysin inhibitors in HF, but no molecule proved to be effective when used alone. This has been attributed to a blunt in Ang-II breakdown by reduced neprilysin activity, increasing Ang-II concentrations. Therefore, neprilysin inhibitors were first tested in combination with ACE inhibitors, as omapatrilat [90]. This combination proved harmful. In fact, both ACE and neprilysin are responsible for the degradation of bradykinin; hence, their concurrent inhibition led to the rise of bradykinin levels with increased risk of angioedema [91]. Conversely, the association of neprilysin inhibitor and ARB proved effective and safe, and represents the only available neurohormonal modulator to date providing both antagonism of RAAS pathway and potentiation of the natriuretic peptide axis. The Prospective Comparison of ARNI with ACEI to Determine Impact on Global Mortality and Morbidity in Heart Failure (PARADIGM) trial was the first study to demonstrate the prognostic benefits of a neprilysin inhibitor (sacubitril) combined with the ARB valsartan over enalapril in a large population with HFrEF [92]. Still, as assessed in the Prospective Comparison of angiotensin receptor–neprilysin inhibitor with ARB Global Outcomes in HF with Preserved Ejection Fraction (PARAGON-HF) trial, the use of sacubitril/valsartan missed the composite primary endpoint of reducing hospitalization and death in HFpEF [93].

## Novel therapeutic targets

### NO synthase and guanylate cyclase

NO is highly volatile molecule with a short half-life that mediates vasodilation in coronary and non-coronary districts and has positive effects on the myocardium [94]. Once NO is produced in the endothelium and in the endocardium, it spreads to the vSMCs and the myocardium and is responsible for soluble GC activation [95]. The resulting cGMP causes a decrease in intracellular free calcium levels, promoting relaxation, and is then degraded by phosphodiesterase type 5 [95–97]. In HF, NO production is significantly reduced because of multiple mechanisms including downregulation of endothelial NO synthase and inactivation by ROS, especially superoxide anions [98]. This is in turn responsible for vasoconstriction, increased muscular, and vascular stiffness and adverse remodeling [96, 99].

A broad range of molecules targeting the NO-GC system has been developed. Phosphodiesterase 5 inhibitors (such as sildenafil) reduce cGMP degradation [100]. Other drugs directly interact with the soluble form of GC and are classified into activators (cinaciguat) and stimulators (vericiguat and riociguat). GC activators target the oxidized, malfunctioning, NO-unresponsive enzyme by mimicking NO itself. Conversely, GC stimulators act on the reduced, functioning isoform, enhancing soluble GC activity in the presence of the endogenous ligand [101]. Benefits of The SOluble Guanylate Cyclase stimulatoR in heArT failurE patientS With REDUCED EF (SOCRATES-REDUCED) trial showed a reduction in NT-proBNP levels with vericiguat in patients with HFrEF [102, 103]. In the recent Vericiguat Global Study in Subjects with Heart Failure with Reduced Ejection Fraction (VICTORIA) trial, vericiguat reduced the composite end-point of death from any cause or hospitalization for HF compared to placebo among patients with HF at high risk of decompensation [104].

### SGLT2 inhibitors

Sodium glucose cotransporter 2 (SGLT2) inhibitors were specifically designed for the management of type 2 diabetes mellitus, as they promote renal glucose excretion by inhibiting reabsorption [105]. Nonetheless, their effects go far beyond their role of glucose-lowering drugs, as they have been proved to be effective in the context of HFrEF, even in the absence of diabetes. Data from large randomized clinical trials with empagliflozin (EMPA-REG, Empagliflozin, Cardiovascular Outcomes, and Mortality in Type 2 Diabetes) [106] and dapagliflozin (DECLARE–TIMI 58, Dapagliflozin and Cardiovascular Outcomes in Type 2 Diabetes) [107] demonstrated a reduction in hospitalization for HF, in cardiovascular and all-cause mortality, in atherosclerosis-related events and in the progression of chronic kidney disease in large cohorts of diabetic patients. These data have been empowered by the results of the EMPEROR-Reduced (EMPagliflozin outcomE tRial in Patients With chrOnic heaRt Failure With Reduced Ejection Fraction) [108] and the DAPA-HF (Dapagliflozin in Patients with Heart Failure and Reduced Ejection Fraction) [109] trials, which demonstrated a lower risk of cardiovascular death and HF hospitalization with empagliflozin and dapagliflozin as compared to placebo, irrespective of the presence of diabetes mellitus. Moreover, a slower decline in renal function was observed in those treated with empagliflozin. This may infer that the effects on cardiovascular outcomes are at least partially mediated by nephroprotection. As a support, the Dapagliflozin in Patients with Chronic Kidney Disease (DAPA-CKD) [110] demonstrated that dapagliflozin reduced the risk of worsening renal function or death from cardiovascular or kidney disease in patients with chronic kidney disease with and without type 2 diabetes mellitus.

The mechanisms underlying the beneficial effects of SGLT2 inhibitors in HF remain to be elucidated, although some hypotheses have been formulated. As recently reported, SGLT2 inhibitors reduce oxidative stress, inflammation, and fibrosis in the whole cardiovascular system, and mitigate glomerular hypertension, thus preventing shear stress-related renal damage [111–114]. Moreover, they reduced cardiac cytosolic Na^+^ and Ca^2+^ concentrations through inhibition of Na^+^/H^+^ exchanger, promote weight loss by inducing a fasting-like state with increased production of ketones, an advantageous substrate for the failing heart [115]. Finally, SGLT2 inhibitors may blunt sympathetic activation, as they reduce renal and cardiac levels of tyrosine hydroxylase and norepinephrine, and their hypotensive effect is maintained regardless of renal function worsening [116].

### Myocardial contractility

So far, inotropic agents have been predominantly employed in the setting of acute HF. Indeed, molecules most commonly used in clinical practice are burdened by a high rate of adverse effects, especially in end-stage patients, and their use should be limited to the shortest time possible. First-generation inotropes (such as adrenergic agonists, e.g., norepinephrine) are affected by a relevant pro-arrhythmic burden, due to increased oxygen consumption and to the afterdepolarizations caused by calcium overload [117]. With these premises, they are not recommended in the absence of hypotension or hypoperfusion [4]. Glycosides can be used also in the setting of chronic HF, even if they are not associated with improved survival [118]. Levosimendan mainly acts as a calcium-sensitizer, has a more favorable safety profile as compared with adrenergic agonists, and has been shown to have beneficial effects in acute HF [119]. Furthermore, encouraging results came from the Levosimendan Intermittent administration in Outpatients: effects on Natriuretic peptides in advanced chronic HEART failure (LION-HEART) trial, where the intermittent use of levosimendan in patients with advanced HFrEF was associated to a reduction in NT-proBNP levels and in the rate of hospitalization [120].

More recently, new molecules named “myosin motor activators” have been developed, the first-in-class drug being omecamtiv mecarbil. This drug enhances the ATPase activity of myosin, thus favoring the formation and stabilization of cardiomyocyte cross-bridges. This results in an increased force of contraction and in a prolongation of the systolic ejection time, without interfering in calcium transients nor in the velocity of contraction [121, 122]. As compared to “conventional” inotropes, omecamtiv mecarbil improves systolic function without affecting the intracellular concentrations of cAMP and calcium, nor increasing oxygen consumption and ATP demand [123]. Phase II and III trials showed promising results in terms of increase in stroke volume and reduction in end-systolic and end-diastolic diameters. Recently, the GALACTIC-HF trial (A Double-blind, Randomized, Placebo-controlled, Multicenter Study to Assess the Efficacy and Safety of Omecamtiv Mecarbil on Mortality and Morbidity in Subjects With Chronic Heart Failure With Reduced Ejection Fraction) has shown that use of omecamtiv mecarbil was associated with a lower incidence of a composite of a HF event or death from cardiovascular causes compared to placebo [124, 125].

### Mitochondria and metabolism

**Fig. 4:**
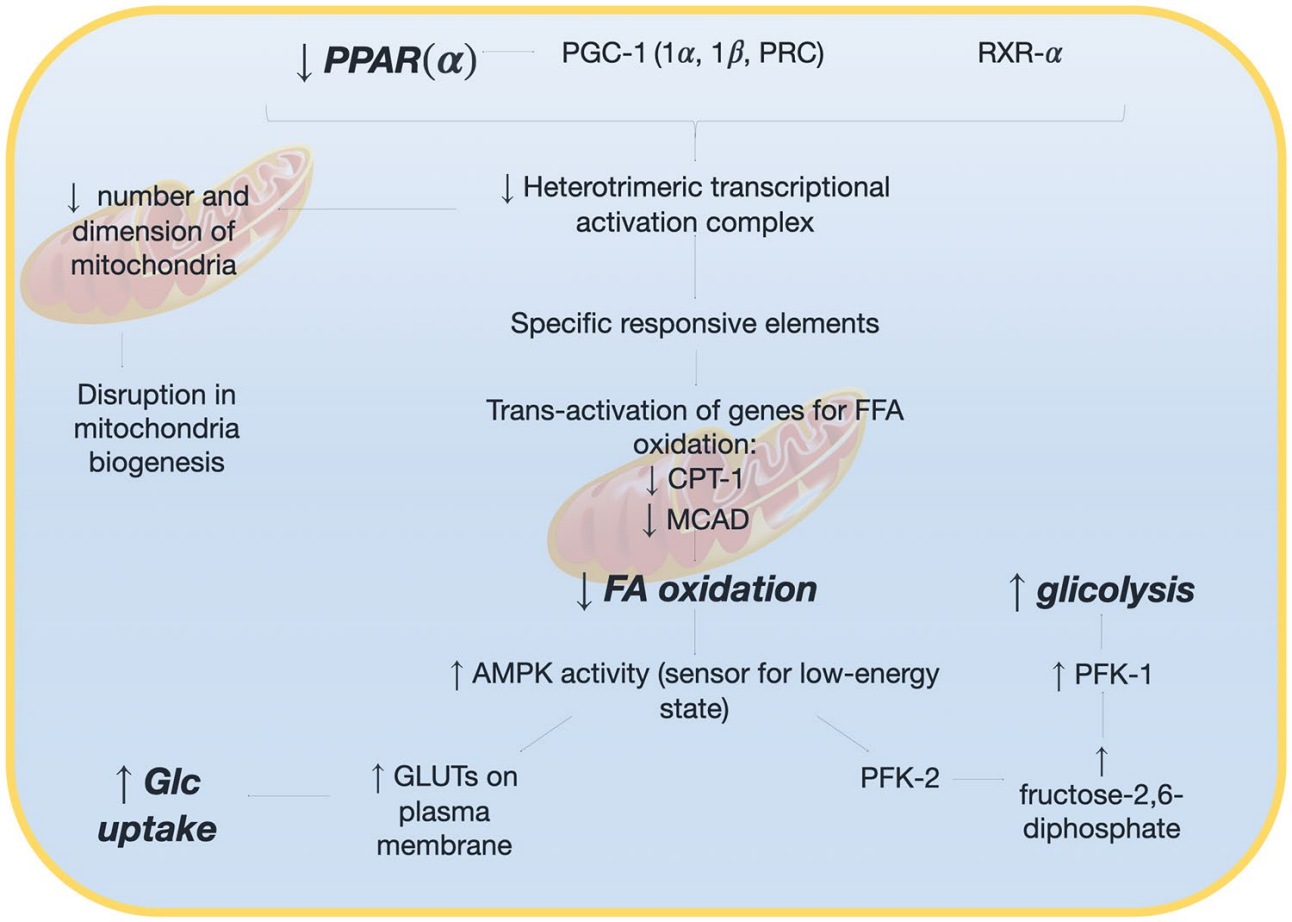
Metabolic phenotype of heart failure. The reduction in peroxisome proliferator-activated receptor alpha (PPAR-$\alpha$) leads to a decreased expression of enzymes for fatty acid oxidation. This in turn stimulates glycolysis and glucose uptake via the increase of adenosine monophosphate kinase. *AMPK* adenosine monophosphate kinase; *CPT* carnitine palmitoyl transferase; *FA* fatty acid; *Glc* glucose; *GLUT* glucose transporter; *MCAD* medium-chain acyl-CoA dehydrogenase; *PFK* phosphofructokinase; *PGC* peroxisome proliferator-activated receptor gamma coactivator 1-alpha; *PPAR* peroxisome proliferator-activated receptor; *RXR* retinoid-X receptor

The resting healthy myocardium drains 60–70% of energy from fatty acid oxidation, whereas energy is mostly obtained from glucose catabolism in post-prandial phase or during physical exercise [126]. Glucose and lipid metabolism are tightly inversely regulated in myocytes according to the Randle cycle [127]. A shift from free fatty acids to glucose utilization has been observed in HF [128]. This mechanism plays an adaptive role, since free fatty acid oxidation requires 10–15% more oxygen to obtain the same ATP levels than glucose. The reduction in peroxisome proliferator-activated receptor (PPAR)-$\alpha$ leads to reduced a number and dimension of mitochondria and a reduced expression of proteins involved in $\beta$-oxidation [129]. Moreover, the increased activity of AMP-kinase, a sensor for low-energy state, promotes the expression of glucose transporters on the plasma membrane and the activity of phosphofructokinase-2, thus increasing glycolysis rate [130] (Fig. 4). Adrenergic activation leads to increased lipolysis from adipose tissue and reactive oxygen species (ROS) production, with an impairment of cell respiration, an upregulation of mitochondrial expression of uncoupling proteins 2 and 3 and insulin resistance [131, 132], finally inhibiting the metabolic switch in the heart.

PPAR-$\alpha$ antagonists might favor this glycolytic shift and improve myocardial energetics, but their clinical utility remains to be established [133]. Trimetazidine, a second-line antianginal drug, promotes a shift to glucose metabolism by inhibiting the last reaction of free fatty acid oxidation, catalyzed by acetyl CoA C-acyltransferase [134]. Growing evidence supports its efficacy in chronic HF, even if most of the available data come from meta-analyses of retrospective studies [135–137].

Mitochondrial function also relies on membrane stability, mainly dependent on cardiolipin and coenzyme Q10 [138, 139]. Elamipretide, a cardiolipin-stabilizer, has been first tested on a canine model of HF showing improvements in LVEF and reduction in circulating levels of NT-proBNP, tumor necrosis factor-$\alpha$ and C-reactive protein. Although no significant reduction in biomarkers emerged from studies in humans, some positive effects on reverse remodeling were observed [140, 141]. Though, a phase-II clinical trial showed no improvement in left ventricular end-systolic volume after a 4-week treatment [142]. Coenzyme Q10 promotes the synthesis of ATP in the mitochondria by participating in redox reactions within the electron transport chain. Treatment with coenzyme Q10, studied in The Effect of Coenzyme Q10 on Morbidity and Mortality in Chronic Heart Failure (Q-SYMBIO) study, was associated with a lower rate of mortality for both cardiovascular- and all-cause mortality and with a reduced rate of hospitalization. However, studies on larger cohorts are still lacking [143].

The impairment in cellular respiration is also associated to enhanced oxidative stress and inflammation. Patients with chronic HF show higher levels of serum biomarkers associated with inflammation, including high-sensitivity C-reactive protein, interleukin-1β, interleukin-6, inteleukin-8, and tumor necrosis factor [144]. An excess in ROS can modify highly expressed proteins in myocytes, including protein-kinases, myofilaments and other proteins involved in excitation–contraction coupling [145]. In a cohort of high-risk coronary artery disease patients, interleukin-1β blockade was associated with significant reduction in ischemic events and cardiovascular mortality [146]. A post hoc analysisalso detected a reduction in the rate of hospitalization due to HF [147].

### Treatments targeting hypertrophy and fibrosis

**Fig. 5:**
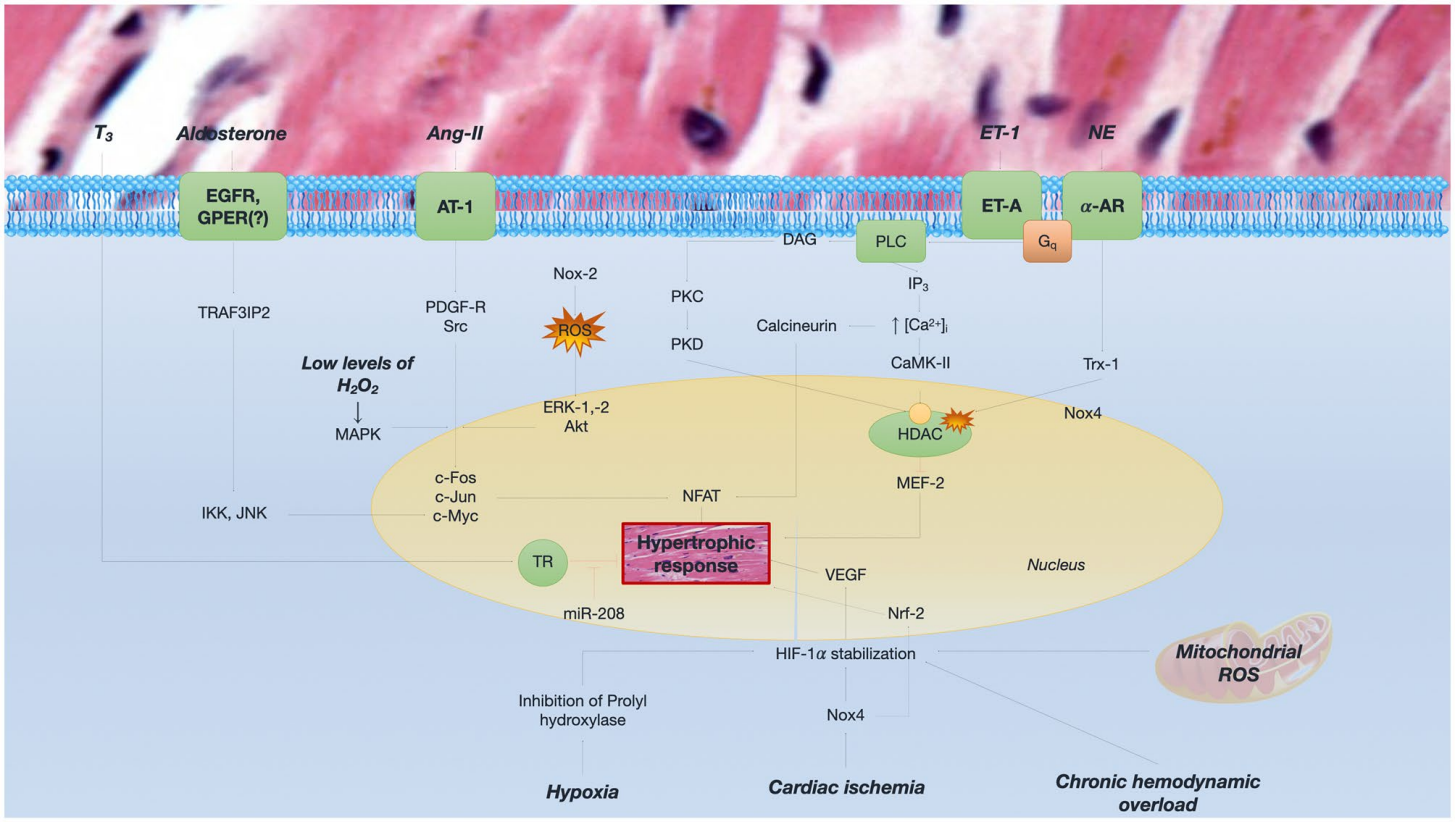
Main molecular pathways for cardiac hypertrophy. Hypertrophy may be the consequence of both hemodynamic (chronic overload) and non-hemodynamic factors, namely neuroendocrine systems derangement and reduction in oxygen myocardial delivery. *Ang* angiotensin; *AR* adrenergic receptor; *AT* angiotensin receptor; *DAG* diacyl-glycerol; *EGFR* epidermal growth factor receptor; *ERK* extracellular signal-regulated kinase; *ET* endothelin; *ET-A* endothelin receptor A; *H*_*2*_*O*_*2*_ hydrogen peroxide; *HDAC* histone deacetylase; *HIF* hypoxia inducible factor; *IKK* inhibitor of nuclear factor kappa-B kinase; *IP3* inositol triphosphate; *JNK* c-Jun N-terminal kinase; *MEF* myocyte enhancer factor; *NE* norepinephrine; *NFAT* nuclear factor of activated T-cell; *Nox* NADPH oxidase; *PDGF-R* receptor of platelet-derived growth factor; *PKC* protein kinase C; *PKD* protein kinase D; *PLC* phospholipase C; *Src* Rous sarcoma protooncogene; *TR* thyroid receptor; *Trx1* thioredoxin 1; *VEGF* vascular endothelial growth factor

Beyond antagonists of renin–angiotensin–aldosterone system and beta-blockers that affect cellular hypertrophy [148], current efforts are focusing on the anti-hypertrophic effects of molecules acting directly on the cardiomyocyte, targeting crucial signaling cascades that alter gene expression, protein function, and red149ox imbalance (Fig. 5). These agents include histone deacetylase inhibitors, a wide spectrum of pro-hypertrophic microRNAs and several other small molecules (such as rapamycin, inhibitors of Rho kinase) [149–151].

In contrast to the traditional view in which fibrosis is regarded as a secondary phenomenon, recent evidences indicate a primary role for cardiac fibroblast activity in myocardial disease. Cardiac fibrosis is linked to cardiac dysfunction, increased risk of arrhythmia, and poor outcomes [152]. Despite the use of ACE inhibitors and MRAs, a residual fibrotic activity is still observed in HF. Novel antifibrotic agents are currently under investigation, specifically targeting connective tissue growth factors (e.g., TGF-$\beta$ by pirfenidone), galectin-3 (e.g., by antisense RNA), matrix metalloproteinases, and cell reprogramming via non-coding RNAs [153].

### HFpEF: a therapeutic challenge

Unlike in HFrEF, neurohormonal antagonists did not reduce mortality in patients affected by HFpEF. However, when excluding cohorts from Georgia and Russia in the Treatment of Preserved Cardiac Function Heart Failure With an Aldosterone Antagonist (TOPCAT) trial, the use of spironolactone significantly reduced mortality in this setting [154]. The finding that aldosterone levels are increased in HFpEF may provide a pathophysiological basis for the use of MRA in HFpEF [155].

To date, treatment of risk factors and underlying disease is the only beneficial therapeutic approach for patients with HFpEF. Notably, tafamidis, a pharmacological transthyretin-stabilizer, improved outcome of patients affected by transthyretin-related cardiac amyloidosis, a progressively more recognized clinical phenotype of HFpEF [156].

## Future perspectives

Novel therapeutic options are emerging for the treatment of HF. Some of them address well-known pathophysiological mechanisms, such as neurohormonal deregulation and the NO-GC-cGMP system; others, such as SGLT2 inhibitors, hold effects on the cardiovascular system that are still to be fully clarified. Several emerging approaches target cardiac metabolism, inflammation, and remodeling. Specific transient receptor potential cation channel subfamily V (TRPV)-4 blocker, GSK2798745, has shown promising results in a model of chronic HF, possibly by interfering with transforming growth factor (TGF) β-1-induced activation of myofibroblasts [157].

In the future of HF therapy, stem cells, micro-RNA, and epigenetics are likely to become novel cornerstone and to allow a tailored approach. Micro-RNAs have been thoroughly studied in the processes of hypertrophy, apoptosis, and fibrosis. As an example, micro-RNAs-34 family encloses many subtypes of micro-RNAs whom are involved in fibroblast survival and growth factors secretion (i.e., micro-RNA-21) [158] and in the activity of calmodulin kinase (i.e., micro-RNA-1) [159]. As shown in the Randomized Clinical Trial of Intravenous Infusion Umbilical Cord Mesenchymal Stem Cells on Cardiopathy (RIMECARD), the use of umbilical cord stem cells was safe in patients with stable HFrEF under optimal medical treatment, and improvements in LVEF, functional status and quality of life were observed [160].

## Conclusions

HF is a complex, multisystemic syndrome with a dramatic social and economic impact. Since decades, HF therapeutics have been following the interpretative pathophysiological models. After initial strategies targeting inotropism and congestion, and neurohormonal antagonism addressing dysregulation of peripheral feedbacks, novel therapeutic approaches have been recently developed, directed to the heart muscle. Drugs acting at a second-messenger levels, such as vericiguat, as well as other drugs acting on myocardial energetics and mitochondrial function, including elamipretide or omecamtiv mecarbil may then represent in the next future some additive, synergistic tools to improve patient outcome. Effective, evidence-based drugs from the fields of stem cells, micro-RNAs, and epigenetic remodulation are awaited in the next decades. In the next future, individual profiling and dissection of the activation of specific pathophysiological pathways of organ damage may represent the base for a tailored treatment of HF.

## Abbreviations

ACE: Angiotensin-converting enzyme
ADH: Antidiuretic hormone/arginine-vasopressin
Ang: Angiotensin
ANP: Atrial natriuretic peptide
BNP: B-type natriuretic peptide
cAMP: Cyclic adenosine monophosphate
cGMP: Cyclic guanosine monophosphate
CNP: C-type natriuretic peptide
GC: Guanylate cyclase
HF: Heart failure
HFmrEF: Heart failure with mid-range ejection fraction
HFpEF: Heart failure with preserved ejection fraction
HFrEF: Heart failure with reduced ejection fraction
MAPK: Mitogen-activated protein kinase
MR: Mineralocorticoid receptor
MRA: Mineralocorticoid receptor antagonists
NADPH: Nicotinamide-adenine dinucleotide phosphate
NO: Nitric oxide
NPR: Natriuretic peptide receptor
NT: N-terminal
PPAR: Peroxisome proliferator-activated receptor
RAAS: Renin-angiotensin-aldosterone system
SGLT: Sodium glucose cotransporter
TGF: Transforming growth factor
TRPV-4: Transient receptor potential cation channel subfamily V member 4
vSMCs: Vascular smooth muscle cells
